# Personal Items

**Published:** 1870-12

**Authors:** 


### Personal Items.

Prof. Charles A. Lee has resigned the Chair of Materia Medica and Hygiene in the Medical Department of the University of Buffalo, and his resignation has been accepted by the Faculty. He has long and ably filled the position, and his resignation was only accepted in consideration of enfeebled health. His wide experience, ripe scholarship, and high professional standing, made him distinguished as a teacher. His place is filled the present session by Prof. N. H. Eastman, of Geneva.

Prof. Sandford Eastman, of Buffalo, has resigned the Chair of Anatomy and Clinical Surgery in the University of Buffalo, on account of impaired health. This resignation has been accepted with great regret at the sad necessity, Prof. Eastman is now in California for his health, where the good wishes of his numerous friends will attend him. Dr. Milton Potter has been appointed as Lecturer on Anatomy, to fill the vacancy made by resignation of Prof. Eastman.

Dr. S. W. Wetmore, of Buffalo, has been invited to Lecture on Anatomy in the Medical Department of the University of Wooster, at Cleveland, Ohio, in the place made vacant by the sudden death of Prof. Jones.

